# YAP contributes to DNA methylation remodeling upon mouse embryonic stem cell differentiation

**DOI:** 10.1074/jbc.RA120.015896

**Published:** 2020-12-06

**Authors:** Fabiana Passaro, Ilaria De Martino, Federico Zambelli, Giorgia Di Benedetto, Matteo Barbato, Anna Maria D’Erchia, Caterina Manzari, Graziano Pesole, Margherita Mutarelli, Davide Cacchiarelli, Dario Antonini, Silvia Parisi, Tommaso Russo

**Affiliations:** 1 Department of Molecular Medicine and Medical Biotechnology, University of Napoli Federico II, Italy; 2 Department of Biosciences, University of Milano, Italy; 3 Institute of Biomembranes, Bioenergetics and Molecular Biotechnologies, National Research Council, Italy; 4 Department of Biosciences, Biotechnology and Biopharmaceutics, University of Bari, Italy; 5 Tigem and Department of Translational Medicine, University of Napoli Federico II, Italy; 6 Department of Biology, University of Napoli Federico II, Italy

**Keywords:** Yes-associated protein, DNA methyltransferases, Dnmt3l, ephemeron, embryonic stem cells, epiblast-like stem cells, differentiation, pluripotency, stemness, AP, alkaline phosphatase, ChIP-Seq, chromatin immunoprecipitation sequencing, CTR KD, control KD, DMRs, differentially methylated regions, ESCs, embryonic stem cells, FACS, fluorescence-activated cell sorting, FC, fold change, GO, gene ontology, KD, knockdown, KO, knockout, LIF, leukemia inhibitory factor, lncRNA, long noncoding, OE, overexpression, SFEBs, serum-free embryoid bodies, TAZ, WW-domain-containing transcription regulator 1 (WWTR1 also known as TAZ), TEADs, Transcriptional Enhanced Associate Domains, YAP, Yes-associated protein

## Abstract

The Yes-associated protein (YAP), one of the major effectors of the Hippo pathway together with its related protein WW-domain-containing transcription regulator 1 (WWTR1; also known as TAZ), mediates a range of cellular processes from proliferation and death to morphogenesis. YAP and WW-domain-containing transcription regulator 1 (WWTR1; also known as TAZ) regulate a large number of target genes, acting as coactivators of DNA-binding transcription factors or as negative regulators of transcription by interacting with the nucleosome remodeling and histone deacetylase complexes. YAP is expressed in self-renewing embryonic stem cells (ESCs), although it is still debated whether it plays any crucial roles in the control of either stemness or differentiation. Here we show that the transient downregulation of YAP in mouse ESCs perturbs cellular homeostasis, leading to the inability to differentiate properly. Bisulfite genomic sequencing revealed that this transient knockdown caused a genome-wide alteration of the DNA methylation remodeling that takes place during the early steps of differentiation, suggesting that the phenotype we observed might be due to the dysregulation of some of the mechanisms involved in regulation of ESC exit from pluripotency. By gene expression analysis, we identified two molecules that could have a role in the altered genome-wide methylation profile: the long noncoding RNA ephemeron, whose rapid upregulation is crucial for the transition of ESCs into epiblast, and the methyltransferase-like protein Dnmt3l, which, during the embryo development, cooperates with Dnmt3a and Dnmt3b to contribute to the *de novo* DNA methylation that governs early steps of ESC differentiation. These data suggest a new role for YAP in the governance of the epigenetic dynamics of exit from pluripotency.

One of the molecular machineries that play crucial roles during embryo development is that involving the Yes-associated protein (YAP) and the related protein WW-domain-containing transcription regulator 1 (WWTR1; also known as TAZ) (1, 2). These two proteins play a fundamental role in the so-called Hippo pathway, as they, through a cytosol–nucleus shuttling regulated by nucleus-excluding phosphorylation, govern the transcription of various genes involved in sensing mechanical stress (3), cell proliferation and apoptosis (4, 5), and organ size (6). YAP and TAZ function as coactivators of the Transcriptional Enhanced Associate Domains (TEADs) (1), but the multitasking ability of YAP/TAZ is demonstrated by many results indicating TEAD-independent functions even outside the nucleus (7).

The critical role of these two proteins during the very early steps of development is recapitulated by the phenotype of YAP/TAZ double knockout (KO): these embryos are arrested in the premorula stage (8), likely because of the induced repression of Sox2 preventing the appearance of the inner cell mass phenotype. At the morula stage, YAP is responsible for the activation of trophoblast master genes, like *Cdx2*, in the cells of the external layer, thus governing the acquisition of the trophoblast cell identity (9). Besides, YAP is expressed in the blastocyst and, *in vitro*, in embryonic stem cells (ESCs). Several works aimed at characterizing the function of YAP/TAZ in ESCs. Some results indicated that the suppression of YAP or TEAD resulted in the decreased intensity of alkaline phosphatase (AP) staining of colonies and the downregulation of Oct4 and Sox2, with concomitant expression of differentiation markers, like T, alpha-fetoprotein, and Gata4 (10). Accordingly, overexpression (OE) of a TEAD dominant-negative protein led to the induction of ESC differentiation toward the endodermal lineage (11). *In vivo* analysis showed that high TEAD activity sustains pluripotency in the inner cell mass, whereas cells with low TEAD levels are eliminated (5). However, conflicting results showed that YAP/TAZ depletion had no effects on the stemness of ESCs grown in 2i medium and that, in these 2i conditions, YAP/TAZ downregulation mimics GSK3 inhibitor that blocks the β-catenin pathway (7). Consistent with these results, the silencing or the KO of YAP has no effects on the maintenance of the undifferentiated state (12). Furthermore, in differentiation-inducing conditions, YAP knockdown (KD) results in an insufficient accumulation of differentiation markers, like T, Gata6, and Gata3, although Oct4 and Nanog are normally suppressed (12).

These apparently conflicting results could at least in part depend on the multifaceted activities of YAP/TAZ in the various steps of ESC differentiation, whose suppression in different experimental conditions could lead to diverse consequences. To address this point, we explored the effects of a very transient downregulation of YAP on the differentiation of mouse ESCs. We found that, although the normal YAP levels are completely rescued during the differentiation process, YAP KD cells show a genome methylation profile significantly different from that of the control cells. Looking at the expression profile of undifferentiated YAP KD cells, we appreciated significant downregulation of Dnmt3l and the ephemeron long noncoding (lncRNA). These findings indicate that YAP expression in undifferentiated ESCs is necessary to sustain the appropriate machinery responsible for the remodeling of the genome methylation patterns taking place at the exit of ESCs from the naive state.

## Results

### Transient KD of YAP affects early steps of differentiation of ESCs

**Figure 1:**
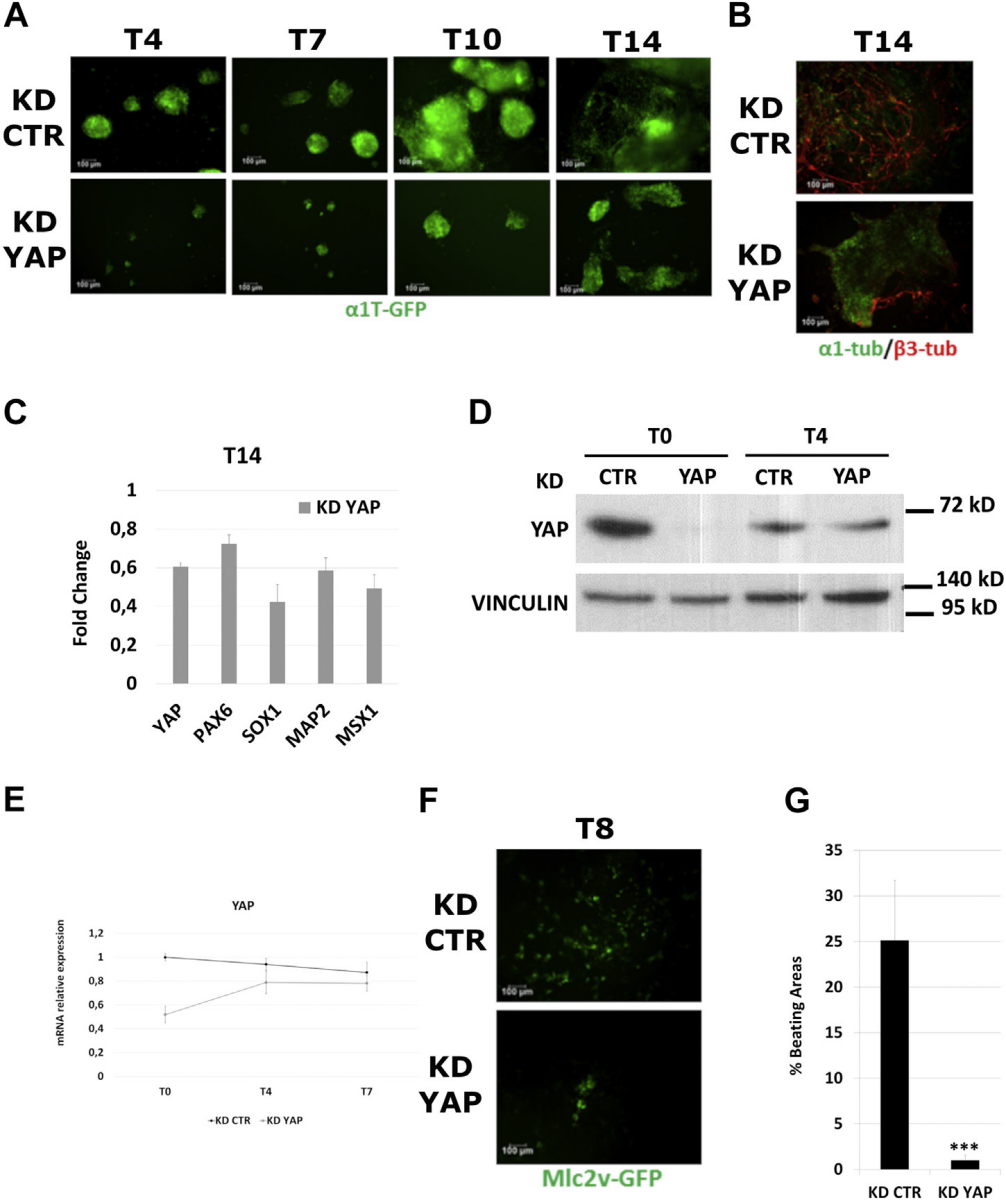
**Transient KD of YAP impairs differentiation of ESCs.***A,* α1T–GFP stable cell line transfected with Stealth siRNA to silence YAP expression (KD YAP) showed a decrease in neuroectodermal differentiation efficacy with respect to KD CTR cells. Representative images are shown. The scale bar represents 100 μm. *B,* representative immunostaining of neural marker β3-tubulin (*red*) in α1T–GFP (*green*) at final stage (T14) of differentiation showing a dramatic reduction in postmitotic neuronal differentiation. The scale bar represents 100 μm. *C,* quantitative PCR analysis of neuronal marker gene expression upon differentiation of YAP KD cells. Data are shown as fold changes with respect to KD CTR cells. ∗*p* < 0.05, ∗∗*p* < 0.01. *D,* YAP expression profile over neuroectodermal differentiation by Western blot or *E,* quantitative PCR. *F,* YAP KD in mlc2v–GFP stable cell line showed a strong reduction of mesodermal differentiation efficiency compared with KD CTR cells. Representative immunostainings are shown. The scale bar represents 100 μm. *G,* percentage of beating areas at final stage of differentiation (T8), demonstrating a significant (∗∗∗*p* < 0.01) decrease in generation of mature cardiomyocytes upon YAP KD. For each data set, averaged numbers from biological triplicates were used for statistics. Error bars indicate mean ± SEM. CTR, control; KD, knockdown; YAP, Yes-associated protein.

We decided to explore the possible long-lasting effects of a transient YAP suppression in ESCs. To this aim, E14Tg2a clones, stably expressing GFP under control of the neural-specific promoter of the α1-tubulin gene (α1T–GFP) (13), were transiently transfected with a mixture of siRNAs eliciting a robust suppression of YAP expression (Fig. S1). About 48 h after transfection, YAP KD cells were induced to differentiate toward the neuroectodermal fate (13), and GFP expression was monitored at various time points during the differentiation process. As shown in Fig. 1*A*, the number of GFP-positive cells was significantly decreased in YAP KD since the very first step of differentiation (day 4), with respect to control KD (CTR KD) cells, transiently transfected with siRNA negative control duplex. Accordingly, at later stages (day 14), the differentiation process in YAP KD was incomplete, with a reduced number of cells expressing β3-tubulin (Fig. 1*B*). The expression profile of late marker genes also confirmed that the transient KD of YAP severely affects neuroectodermal differentiation (Fig. 1*C*).

The expression profile of YAP over differentiation steps confirmed the transiency of YAP silencing, as its mRNA and protein levels returned at steady state at day 4 of differentiation (Fig. 1, *D* and *E*). Nevertheless, the consequences of YAP suppression lasted until the end of the differentiation protocol, strongly suggesting that YAP could play a role in ESCs or in the early steps of their differentiation process and that this activity is determinant for the following events.

At this point, we explored the ability of YAP KD cells to differentiate toward the mesendodermal fate to address whether the transient downregulation of YAP expression in ESCs interferes specifically with the pathway of neuroectodermal differentiation or, instead, induces a general perturbation of the differentiation potential of ESCs. To this aim, we transiently transfected with YAP siRNAs, the E14Tg2a clone MLC2v–GFP, in which the expression of GFP is under control of the cardiomyocyte-specific promoter of *MLC2v* gene (14). About 48 h after transfection, YAP KD cells were induced to differentiate into mesodermal derivatives by embryoid bodies formation in mesodermal culture conditions (15). Again, 8 days after the induction of differentiation, the number of GFP-positive cells was reduced in YAP KD cells (Fig. 1*F*), and, interestingly, although cardiomyocyte colonies from CTR KD cells showed the expected beating phenotype, almost no beating areas were found in YAP KD cells (Fig. 1*G*). These results suggest that the transient downregulation of YAP expression compromises the potential of undifferentiated ESCs to undertake differentiation properly.

### Transient KD of YAP causes a perturbation of homeostasis ESCs

The partial inability of ESCs to undertake differentiation could be due, on the one hand, to premature loss of pluripotency or, on the contrary, to the persistence of stemness despite and after the induction of differentiation.

**Figure 2:**
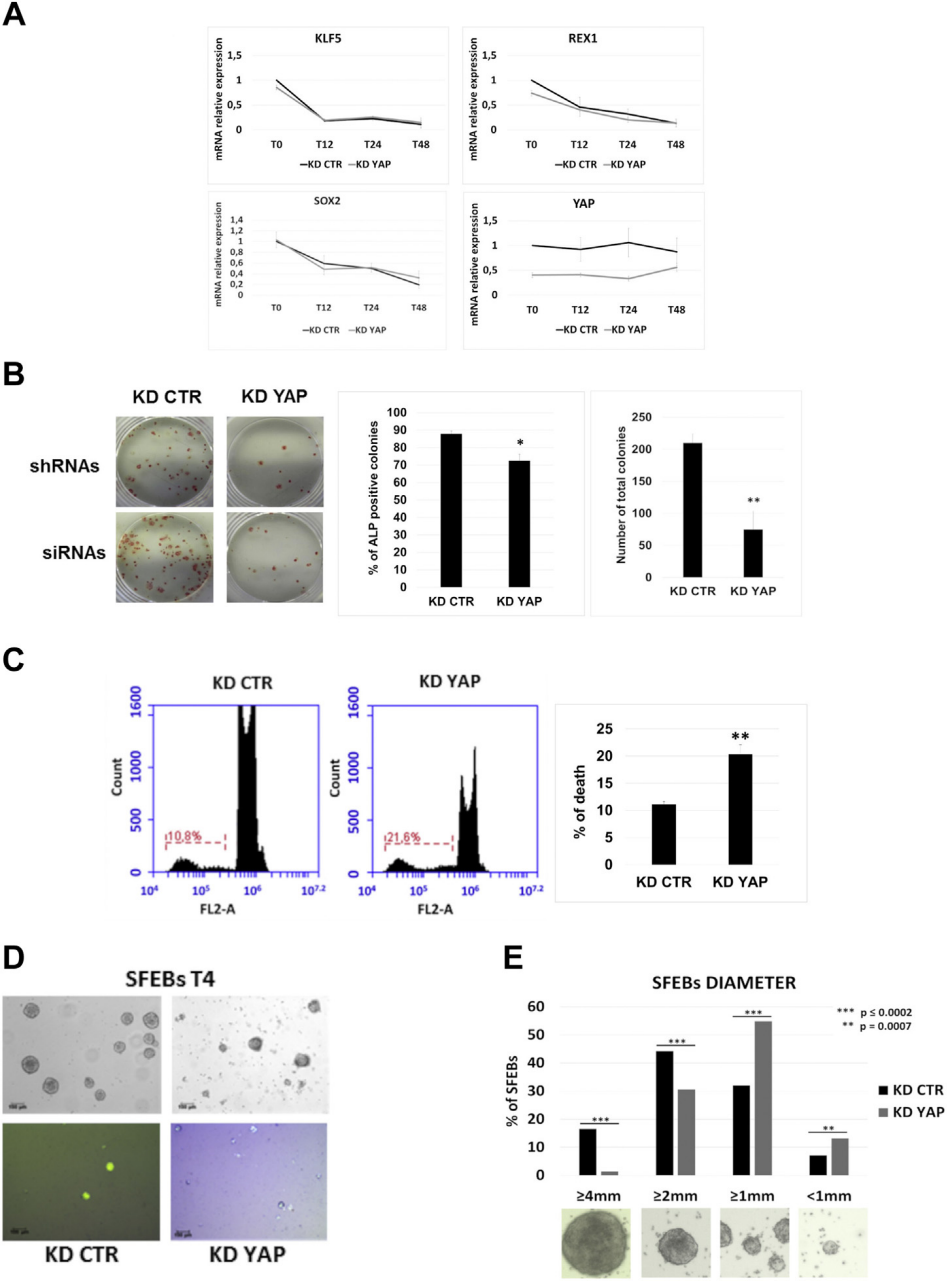
**The KD of YAP does not impair the pluripotency of ESCs but decreases cell viability.***A,* quantitative PCR analysis of stemness marker genes upon leukemia inhibitory factor withdrawal in KD YAP compared with KD CTR cells. For each data set, n = 3. Error bars indicate mean ± SEM. *B,* representative images of alkaline phosphatase (AP) staining at day 7 upon transfection of siRNAs. YAP KD causes a significant (∗∗*p* < 0.01) decrease in the number of total AP colonies, whereas the percentage of AP-positive colonies seems to be almost unaffected (∗*p* < 0.05) with respect to CTR KD cells. For each data set, n = 3. Error bars indicate mean ± SEM. *C,* representative flow cytometry histograms of gated nuclei fluorescence (propidium iodide staining) detected using the FL2 (480 nm) photodetector (FL2-A). YAP KD significantly (∗∗*p* < 0.01) increases the percentage of sub G0/G1 cell population, compared with KD CTR, n = 3. Error bars indicate mean ± SEM. *D,* representative images of KD CTR and KD YAP SFEBs tested with trypan blue exclusion assay. The scale bar represents 100 μm. *E,* statistical analyses of the dimensions of SFEBs from YAP KD compared with CTR KD cells. Diameter measurement was performed using ImageJ 1.52v software. For each data set, 300 SFEBs from each biological triplicate were analyzed. Error bars indicate mean ± SEM. CTR, control; KD, knockdown; SFEBs, serum-free embryoid bodies; YAP, Yes-associated protein.

To explore whether YAP plays some role in the maintenance of the undifferentiated state of ESCs, we transiently silenced YAP expression in E14Tg2a cells by siRNAs or shRNAs (Fig. S2*A*). After 48 h, KD cells were plated in the absence of leukemia inhibitory factor (LIF) for a further 48 h, and RNA samples were analyzed to measure the expression of stemness marker genes. The results shown in Fig. 2*A* demonstrate that the expression profile of the main stemness markers was not affected by the transient silencing of YAP. These cells were also plated at low density and cultured for 7 more days in the presence of LIF and serum to examine the formation of AP+ colonies. As shown in Fig. 2*B*, both CTR and YAP KD cells were able to form AP+ colonies with a robust AP staining. However, a difference emerged in the total number of colonies derived from YAP KD cells, which was significantly lower than that of CTR KD (Fig. 2*B*).

Fluorescence-activated cell sorting (FACS) analysis showed a certain degree of cell death 48 h after YAP silencing (Fig. 2*C*) but not so high to support the possibility that the observed decreased number of AP colonies and differentiated cells were exclusively because of cell death.

To assess YAP KD cell viability during the early steps of differentiation, YAP KD cells were induced to differentiate toward neuroectodermal fate by the formation of serum-free embryoid bodies (SFEBs) (15). Trypan blue exclusion assay showed an increased number of nonvital cells among those in YAP KD SFEBs with respect to CTR KD SFEBs (Fig. 2*D*), which were probably responsible for the differences in the size of SFEBs as assessed by measuring the diameter of SFEBs (Fig. 2*E*), with YAP KD SFEBs appearing smaller than CTR KD SFEBs.

Altogether, these results suggest that transient YAP silencing in undifferentiated ESCs causes a perturbation of cellular homeostasis leading to a reduced survival of cells. The latter can in part explain the decrease in the number of total cells when they are replated to examine self-renewal or differentiation. Nevertheless, surviving cells seem to be unable to differentiate properly, suggesting that the transient downregulation of YAP stably affect the signature of selected subpopulations of surviving cells.

### Bisulfite genomic sequencing reveals a general perturbation in the methylation pattern of YAP KD cells upon differentiation

**Figure 3:**
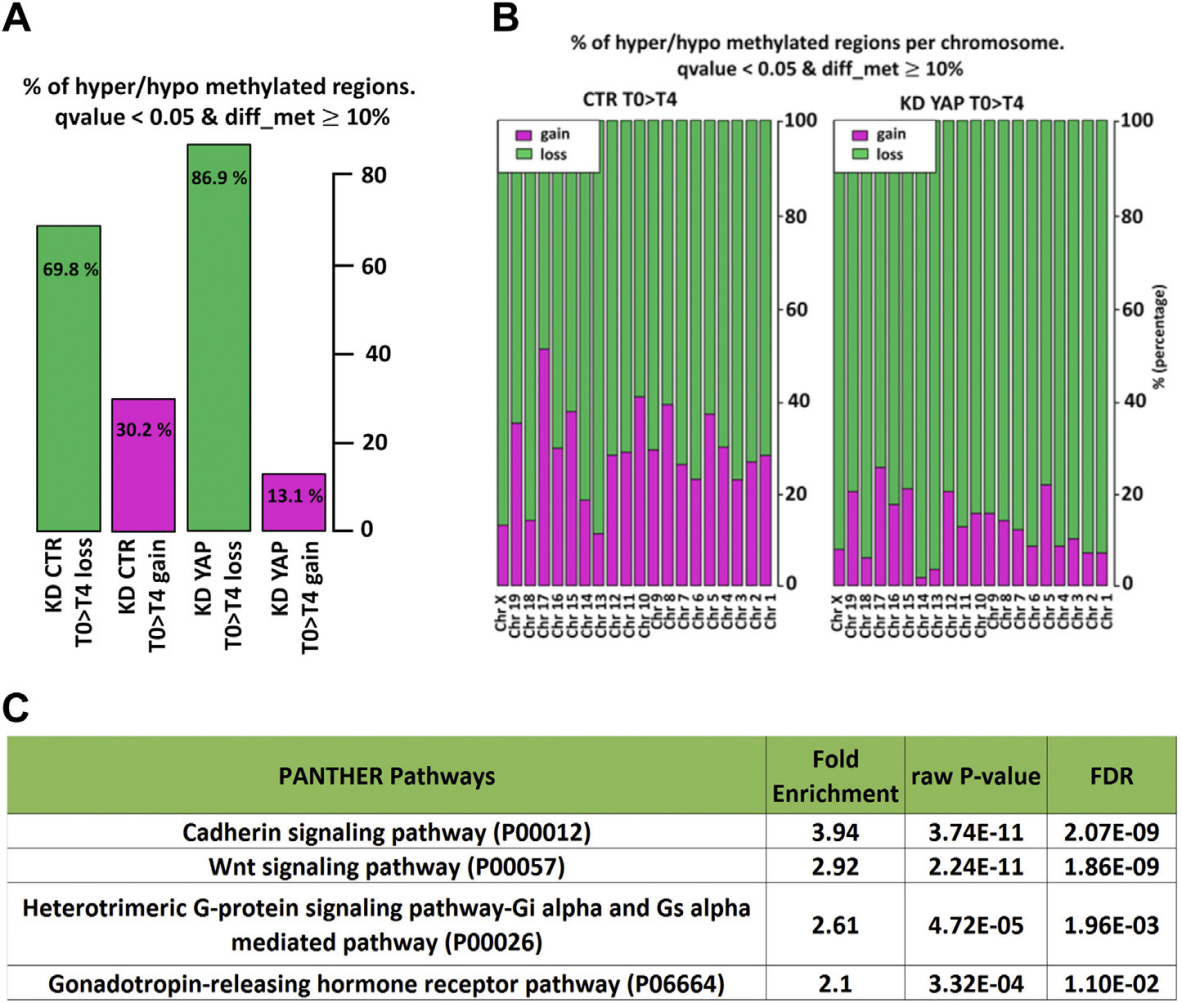
**BS-Seq revealed an imbalance in the methylation pattern of YAP KD cells.***A,* analysis of DMRs at day 4 (T4) of differentiation of serum-free embryoid bodies (SREBs) compared with undifferentiated (T0) cells. Histograms represent the percentage of global loss (*green bar*) and gain (*pink bar*) of methylated regions measured in CTR or YAP KD cells, with a *q* value cutoff of <0.05 and a differential methylation cutoff of >10%. *B,* analysis of distribution of DMRs per chromosome. Histograms represent the percentage of loss (*green bar*) and gain (*pink bar*) of methylated regions per chromosome measured in CTR KD and YAP KD cells at T4 of differentiation of SREBs. *q* Value of <0.05 and differential methylation of >10%. *C,* functional enrichment analysis for significantly over-represented pathways (false discovery rate <0.05) among genes showing a loss of methylation upon YAP KD cells at T4 of differentiation of SFEBs, according to PANTHER. CTR, control; KD, knockdown; PANTHER, protein analysis through evolutionary relationships; YAP, Yes-associated protein.

The phenotypes induced by the transient suppression of YAP are likely because of events that continue to exert their effects also after normal levels of YAP expression are restored. One possibility is that transient YAP silencing could be responsible for deregulation in genome-wide *de novo* methylation, which is known to take place upon the induction of ESC differentiation (16, 17). To explore this possibility, we analyzed by bisulfite sequencing (BS-Seq) the methylation pattern of CTR and YAP KD cell in undifferentiated ESCs (T0) *versus* cells differentiated as SFEBs (T4). First of all, we analyzed the changes in genomic DNA methylation occurring in CTR cells upon 4 days of differentiation, compared with CTR cells at T0, finding 6661 differentially methylated regions (DMRs) with a *q* value ≤0.05 (Table S1). Then, we performed the same analysis in YAP KD cell at T4 SFEBs with respect to T0, identifying 6898 DMRs (Table S1). Although the number of DMRs was similar in absolute terms between the two conditions, the transient suppression of YAP had a dramatic effect on genome methylation. As shown in Fig. 3*A*, about 30% of the 6661 DMRs observed in the differentiation of CTR cells led to a gain of methylation, compared with only 13% gain of methylation in the 6898 DMRs observed in YAP KD cells, whereas, on the other hand, most of the DMRs observed in YAP KD cells were loss of methylation (86.9% *versus* 69.8% in CTR cells), with some chromosomes particularly affected, as in the cases of chromosomes 1, 2, 3, 4, 13, and 14 (Fig. 3*B*).

We then examined the loci showing differences between CTR and YAP KD cells and in particular the loci with differentiation-dependent loss of methylation in YAP KD cells and not in CTR cells and gain of methylation in CTR cells and not in YAP KD cells (Table S1). Considering DMRs with a *q* value of at least 0.05, there are 2325 loci where the loss of methylation was evident only in YAP KD cells and not in CTR cells and 1218 loci where, on the contrary, there is a gain of methylation in CTR cells and not in YAP KD cells. We retrieved the sets of genes closest to the DMRs and analyzed them by using the protein analysis through evolutionary relationships classification system platform for gene ontology (GO) (18). Interestingly, we observed very significant overrepresentation of the Wnt signaling pathway, and the related cadherin pathway, for those genes where the loss of methylation emerged only in YAP KD cells (Fig. 3*C*).

These data demonstrated that the transient downregulation of YAP affects the repatterning of *de novo* methylation occurring in the very early steps of the differentiation of ESCs. This might ultimately lead to the lack of appropriate governance of the epigenetic dynamics occurring at the exit from pluripotency.

### Gene expression profile of YAP KD cells reveals a signature related to *de novo* DNA methylation

**Figure 4:**
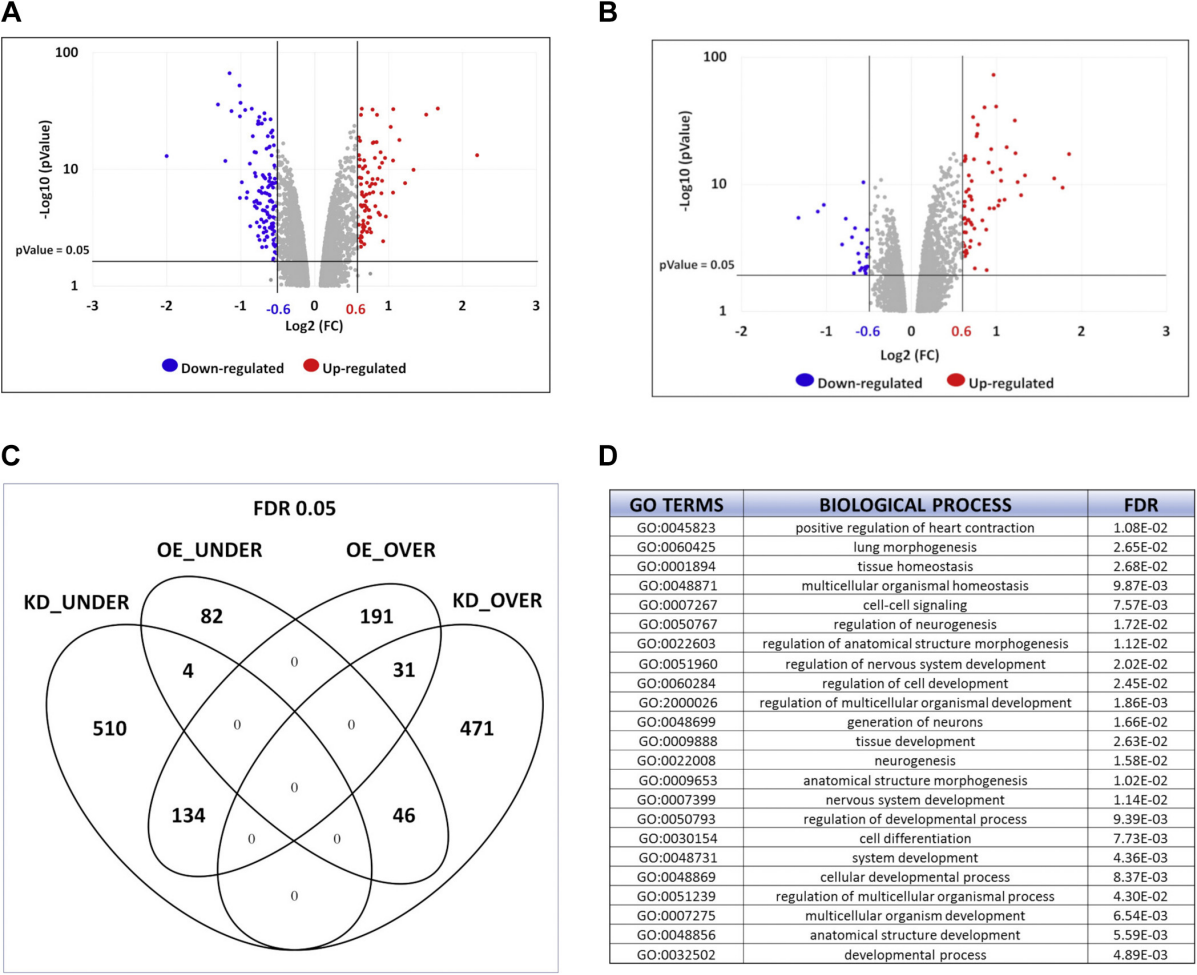
**Gene expression profile of YAP KD cells.***A,* volcano plot for differentially expressed genes (DEGs), which revealed 1196 DEGs in KD YAP cells compared with KD CTR cells. The negative log of *p* value (base 10) is plotted on the *y*-axis, and the log of the FC (base 2) is plotted on the *x*-axis. *Red* plots represent significant (*p* < 0.05) and remarkable (FC > 1.5) upregulated genes, whereas *blue* plots represent significant (*p* < 0.05) and remarkable (FC < 0.7) downregulated genes. *B,* volcano plot showing 488 DEGs in OE YAP cells compared with OE CTR. The negative log of *p* (base 10) is plotted on the *Y*-axis, and the log of the FC (base 2) is plotted on the *X*-axis. *Red* plots represent significant (*p* < 0.05) and remarkable (FC > 1.5) upregulated genes, whereas *blue* plots represent significant (*p* < 0.05) and remarkable (FC < 0.7) downregulated genes. *C,* Venn diagram showing the number of common and unique genes, upregulated (OVER) and downregulated (UNDER), in YAP KD and YAP OE cells. *D,* PANTHER functional enrichment analysis of the most deregulated genes (0.6 < FC > 1.7) in YAP KD for significantly over-represented biological process (FDR < 0.05). GO, gene ontology; KD, knockdown; OE, overexpression; PANTHER, protein analysis through evolutionary relationships.

Considering that the phenotype we observed is caused by a transient downregulation of YAP, we decided to look at the expression profiles of ESCs 48 h after the transfection with YAP siRNAs, when YAP reaches the minimum levels. Total RNA was isolated from three independent samples each for YAP KD and CTR KD and analyzed by RNA-Seq. As shown in Fig. 4*A*, the expression of 1196 genes was significantly deregulated in YAP KD cells (*p* < 0.005; false discovery rate <0.05), with 54% of which resulted in downregulation (Table S2). In parallel, we also analyzed the gene expression profile of ESCs transfected with a YAP-encoding vector (OE YAP) or with the empty vector (OE CTR) (Fig. 4*B*), again using three independent biological samples. In this case, 488 transcripts were found overexpressed (73%) or underexpressed (27%), as a consequence of YAP ectopic expression (Table S2 and Fig. S3).

The comparison between the KD and OE data sets showed that 215 genes were common to both sets, with 134 coherently downregulated in YAP KD cells and upregulated in YAP OE cells, and 46 showing the opposite behavior (Fig. 4*C*).

Protein analysis through evolutionary relationships analysis of the most deregulated genes (0.6 < fold change [FC] > 1.7) in YAP KD cells revealed a statistically significant enrichment (false discovery rate < 0.05) in GO terms related to development and morphogenesis (Fig. 4*D*).

**Figure 5:**
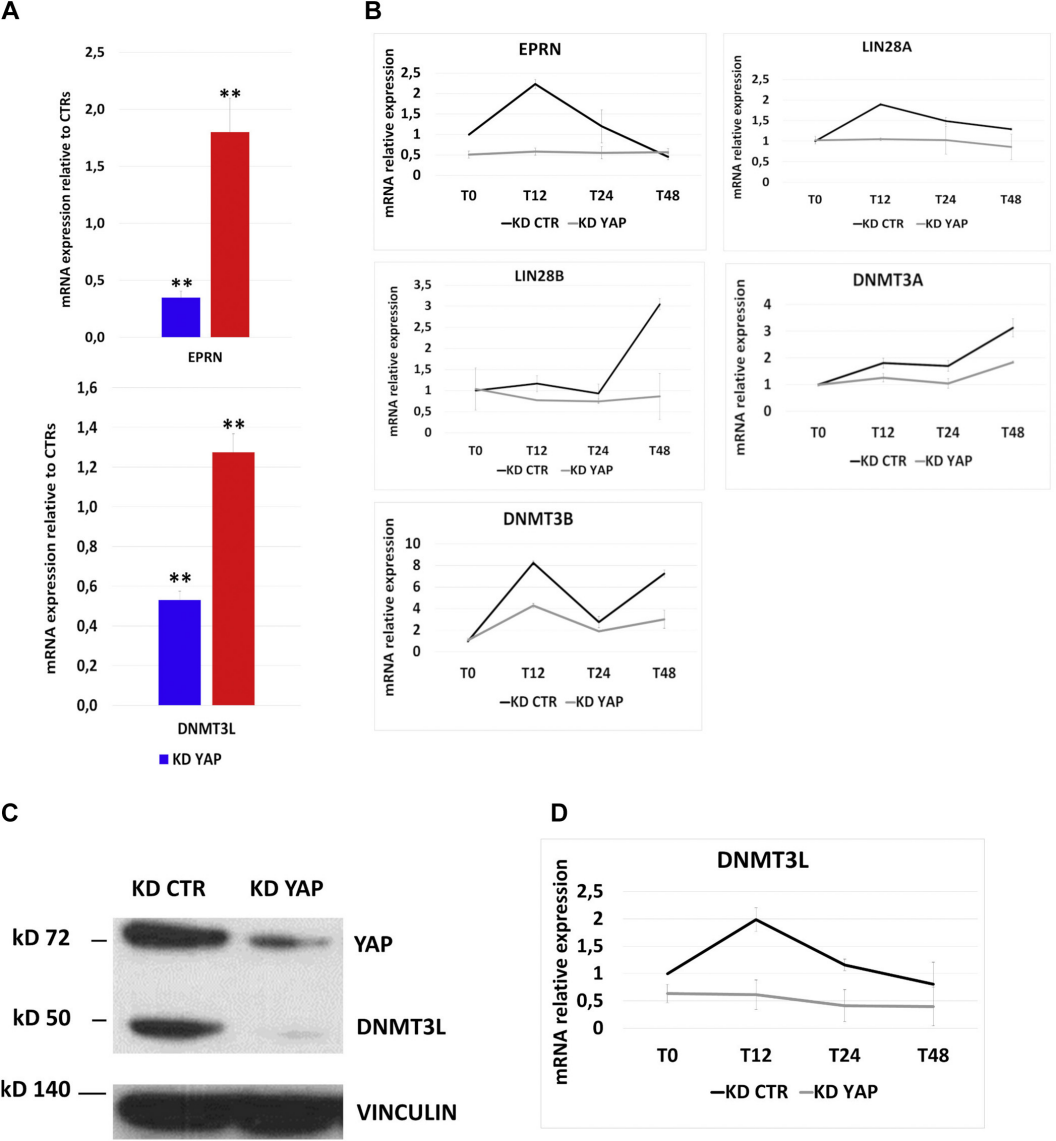
**YAP regulates Eprn and Dnmt3l gene expression.***A,* validation by qPCR analysis of Eprn and Dnmt3l expression upon YAP KD or YAP OE, with respect to CTR cells. For each data set, n = 3. Error bars indicate mean ± SEM. *B,* gene expression profile of Eprn, Lin28a, Lin28b, Dnmt3a, and Dnmt3b in YAP KD cells at different time points (T0–T12–T24–T48 h) upon leukemia inhibitory factor withdrawal. For each data set, n = 3. Error bars indicate mean ± SEM. Data are reported with respect to gene expression profile in KD CTR cells at same time of differentiation. *C,* representative Western blot showing the downregulation of Dnmt3l at protein level upon 48h of YAP KD. *D,* gene expression profile of Dnmt3l in YAP KD cells at different time points (T0–T12–T24–T48 h) upon leukemia inhibitory factor withdrawal. For each data set, n = 3. Error bars indicate mean ± SEM. Data are reported with respect to gene expression profile in KD CTR cells at same time of differentiation. CTR, control; KD, knockdown; YAP, Yes-associated protein.

Among the genes downregulated upon YAP KD and upregulated in YAP OE cells, we focused our attention on two genes that could have a role in the altered genome-wide methylation profile we observed in YAP KD cells. The first one is ephemeron (Eprn—D630045M09Rik), which encodes an lncRNA that modulates the dynamics of exit from naive pluripotency (19). Indeed, it was recently described that Eprn is expressed in undifferentiated mouse ESCs, and its rapid upregulation is crucial for transition of ESCs into Epiblast (19). Upon removal of 2i and LIF, Eprn deletion delays the extinction of ESC identity by reducing Lin28a expression, with the consequent persistence of let-7 microRNAs. In parallel, the upregulation of *de novo* methyltransferase Dnmt3a/b is delayed, which retards embryonic stem cell transition (19). Accordingly, our expression profile of YAP KD cells showed a slight but significant (*p* = 0.0042) decrease of Lin28a (Table S2). We first confirmed that, in our experimental conditions, Eprn was significantly downregulated in undifferentiated ESCs (Fig. 5*A*). We also observed that, although Eprn was induced in CTR cells 12 h after LIF removal, this induction was abolished entirely in YAP KD cells (Fig. 5*B*). In addition, the expression profiles of Lin28a and Lin28b, following LIF withdrawal, were altered in YAP KD cells, with an evident downregulation of Lin28a at T12 and Lin28b at T48. Although not statistically significant, we observed lower mRNA levels also for Dnmt3a and Dnmt3b in YAP KD cells *versus* CTR cells (Fig. 5*B*).

Although RNA-Seq data and quantitative PCR (qPCR) assays did not show significant changes in the expression of Dnmt3a or b, we found that their activating cofactor, Dnmt3l, was downregulated upon YAP KD and upregulated in YAP OE cells. Dnmt3l encodes a protein that, although lacking enzyme activity, cooperates during the embryo development with Dnmt3a and Dnmt3b, contributing to the *de novo* DNA methylation that governs the early steps of ESC differentiation (20). Of interest, Dnmt3l is also expressed in undifferentiated ESCs (21), where it hampers the methylation of bivalent gene promoters by interacting with the polycomb repressive complex 2 (22). We confirmed by qPCR that Dnmt3l was downregulated in ESCs by YAP KD and upregulated upon YAP OE (Fig. 5*A*). Accordingly, Dnmt3l protein was significantly decreased in YAP KD cells (Fig. 5*C*).

As in the case of Eprn, the expression of Dnmt3l was induced 12 h after LIF withdrawal. However, this induction is not observed in YAP KD cells, where Dnmt3l levels remained very low during all the differentiation process (Fig. 5*D*).

### Eprn gene is a direct target of TEAD/YAP ESCs

We addressed the question of whether Dnmt3l and/or Eprn are direct targets of YAP. In the experimental conditions we explored, the fraction of ESCs where YAP is clearly present in the nucleus is relatively low (about 20%), whereas nuclear YAP is undetectable in most, if not all, cells upon the induction to differentiation (Fig. S4, *A* and *B*). Thus, most of the nuclear effects of transient YAP KD should take place in the undifferentiated cells and/or in the very early steps of differentiation. To address this point, chromatin immunoprecipitation sequencing (ChIP-Seq) experiments for YAP were run in duplicate in undifferentiated ESCs. We identified 428 *bona fide* peaks (Table S3) whose transcription start site is located within 500 kbps from the peaks (Fig. S5*A* and Table S3). GO analysis revealed that putative target genes could be significantly clustered based on biological functions associated with epigenetic regulation of gene expression, chromatin silencing, as well as chromatin assembly and disassembly (Fig. S5*B*).

**Figure 6:**
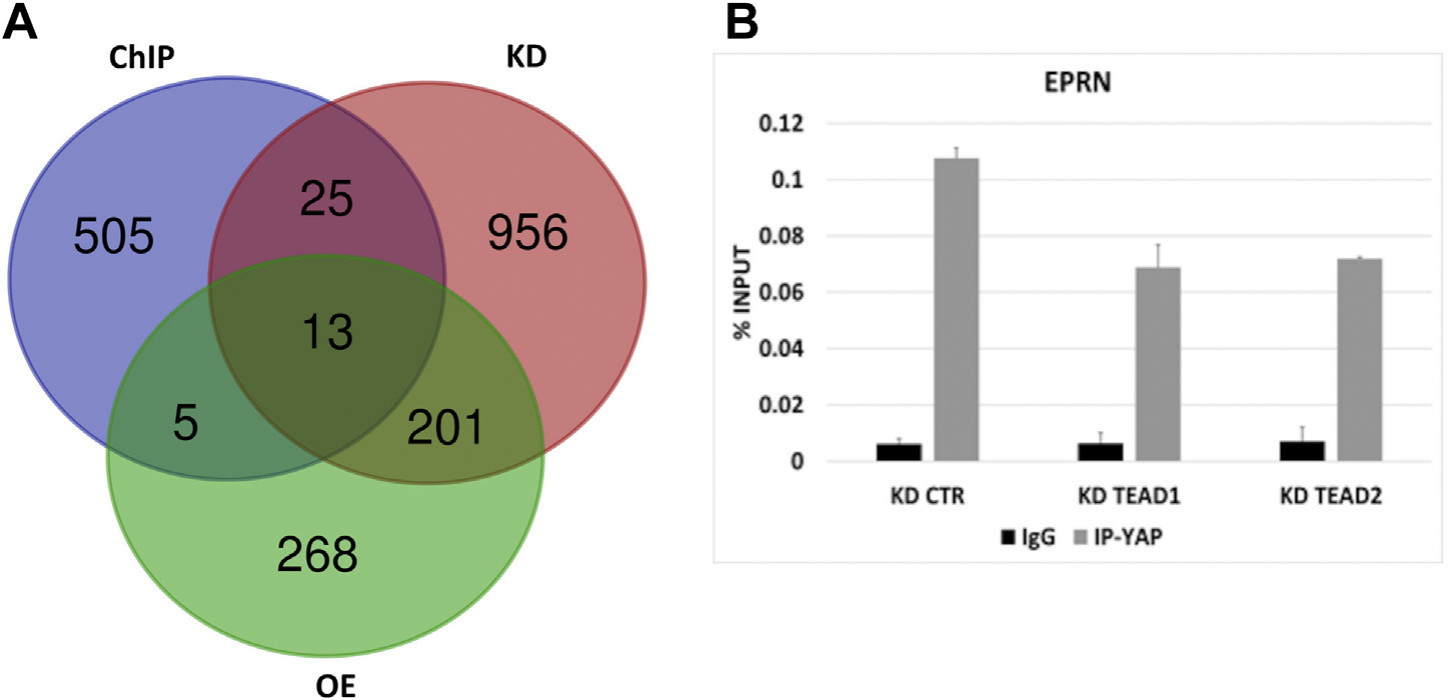
**Eprn is a direct target of YAP/TEAD2.***A,* Venn diagram showing the number of common and unique genes among YAP KD, YAP OE, and YAP ChIP-Seq. *B,* ChIP-qPCR for YAP on Eprn locus in KD CTR, KD TEAD1, and KD TEAD2 cells. Data are shown as percent of precipitated DNA, calculated relative to the total input chromatin, and expressed as the fold enrichment relative to total input. Averaged numbers from biological duplicates were used for statistics. CTR, control; EPRN, ephemeron; KD, knockdown; TEAD, transcriptional enhanced associate domain; YAP, Yes-associated protein.

By crossing ChIP-Seq with RNA-Seq data, we found 43 putative direct targets (Fig. 6*A*), which were deregulated by YAP KD or OE and located in the proximity (500 kbps) of significant ChIP peaks. Neither *Dnmt3l* nor *Eprn* genes showed significant peaks in the proximity of their transcription start site. However, relaxing the stringency threshold, we identified a peak in the second intron of *Eprn* gene (Fig. S6*A*).

About 154 of 428 peaks from our data correspond to TEAD peaks present in published ChIP-Seq collections (23) (Table S4), thus we decided to examine whether TEAD transcription factors could interact with YAP in the direct binding to the *Epnr* gene by ChIP assay, upon silencing of either TEAD1 or TEAD2 (Fig. S5*C*). As shown in Fig. 6*B*, we confirmed the binding of YAP to the peak in the second intron of *Eprn* gene. Moreover, the comparison between the amount of DNA immunoprecipitated in TEADs KD samples with respect to CTR KD revealed that this binding was TEAD dependent. The interaction between YAP and TEADs was confirmed also for a subset of selected peaks from our ChIP-Seq (Fig. S6*B*).

### Dnmt3l and Eprn transient KD affect the differentiation of ESCs

**Figure 7:**
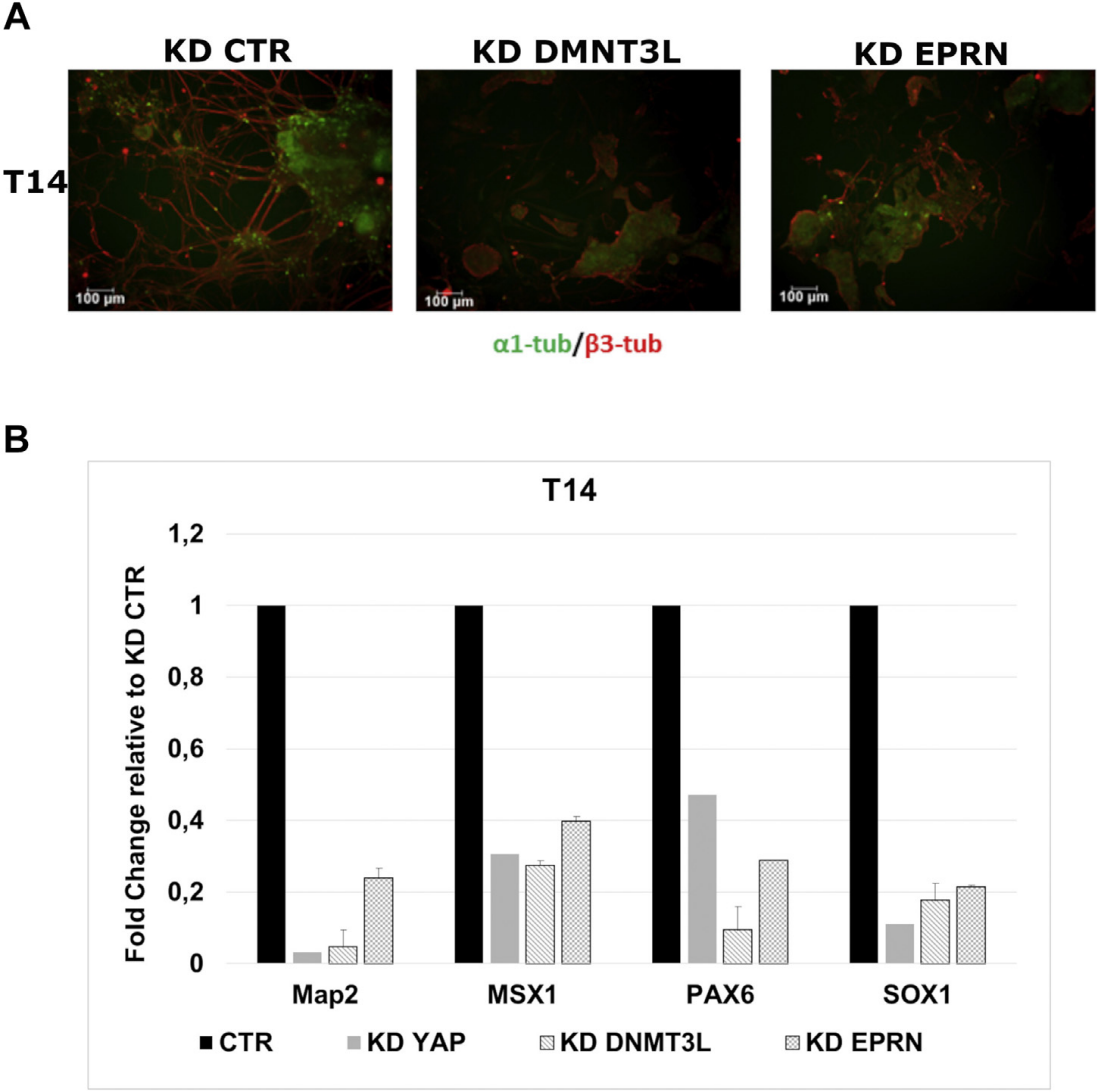
**Eprn and Dnmt3l KD affect the differentiation of ESCs.***A,* α1T–GFP stable cell line transfected with Stealth siRNA to silence YAP (KD YAP), DNMT3L (KD DNMT3L), or EPRN (KD EPRN) expression showed a decrease in neuroectodermal differentiation with respect to KD control cells. Representative immunostaining of neural marker β3-tubulin (*red*) in α1T–GFP (*green*) cells at final stage (T14) of differentiation are shown. The scale bar represents 100 μm. *B,* quantitative PCR analysis of neuronal marker gene expression upon differentiation of KD cells. Data are shown as fold changes with respect to KD CTR cells. For each data set, n = 2. Error bars indicate mean ± SEM. CTR, control; EPRN, ephemeron; KD, knockdown; YAP, Yes-associated protein.

In order to define the contribution of Dnmt3l and Eprn to the phenotype induced by YAP downregulation, α1T–GFP cells were transiently transfected with siRNAs producing a robust suppression of YAP, Dnmt3l, or Eprn expression (Fig. S7*A*). Two independent siRNAs for each target gene were used. Then, 48 h after transfection, KD cells were induced to differentiate toward the neuroectodermal fate. As shown in Fig. 7*A*, both Dnmt3l and Eprn transient suppression lead to a phenotype similar to that induced by YAP downregulation, indeed showing decreased number of cells expressing β3-tubulin at final stage of differentiation. Accordingly, the expression profile of late neural marker genes also confirmed that the downregulation of Dnmt3l or Eprn expression severely affects neuroectodermal differentiation (Fig. 7*B*).

To explore the possibility that the forced expression of a single YAP target could be sufficient to rescue the YAP KD phenotype, we cloned the coding sequence of mouse *Dnmt3l* gene into the pCAG-3xflag vector (13) in order to overexpress the methyltransferase in YAP KD α1T–GFP cells (Fig. S7*A*). About 48 h after transfection, cells were seeded in neuroectodermal differentiation conditions to investigate the effects of Dnmt3l overexpression on neuroectodermal differentiation ether in CTR KD or YAP KD cells. By the evaluation of β3-tubulin–expressing cells at final stage of differentiation, we found that no significant recovery of differentiation functions affected by YAP KD could be obtained by the sole forced expression of Dnmt3L (Fig. S7*B*). Accordingly, no difference was observed in the expression profile of late marker genes in YAP KD cells, with or without Dnmt3l overexpression (Fig. S7*C*).

## Discussion

Here we show results indicating that YAP is required in ESCs to allow them to undergo the epigenetic changes that are necessary for the exit from the undifferentiated state. Indeed, we observed that transient downregulation of YAP affects the changes in the DNA methylation pattern between undifferentiated and differentiated mouse ESCs. This phenotype is characterized by a significant increase in the proportion of regions where methylation is lost and a concomitant decrease in the proportion of regions where *de novo* methylation occurs. In particular, we observed that there are many loci that, upon the exit from pluripotency state, were significantly losing DNA methylation only in YAP KD cells, and that, on the other hand, there are numerous loci where a gain of methylation was only observed in CTR cells. These observations support the conclusion that transient suppression of YAP in ESCs leads to a dysregulation of DNA methylation in terms of both a defect in *de novo* methylation and an excess of demethylation. These phenomena appear to be rather specific because of the very high degree of inappropriate demethylation or lack of *de novo* methylation at specific loci. This observation is further supported by the overrepresentation of genes involved in the Wnt signaling pathway associated with the DMRs. It is worth noting that we observed that more than 20% of genes whose expression is modified as a consequence of transient YAP KD are also putative direct targets of Tcf3 (24). The crosstalk between YAP/TAZ and the Wnt pathway was reported several years ago (25). Indeed, YAP/TAZ regulate, in a transcription-independent fashion, the Wnt pathway by favoring the degradation of β-catenin (7). Our observations point to a possible second YAP-dependent mechanism to regulate the Wnt signaling, based on the methylation, and in turn suppression, of several Wnt-related genes. On the basis of our results, it cannot be excluded that TAZ could contribute to the YAP-dependent mechanisms.

By analyzing the expression profile of ESCs where YAP was transiently downregulated, we realized that this phenotype could be, at least in part, explained by the YAP KD–dependent downregulation of Dnmt3l and lncRNA Eprn. Dnmt3l is similar to the two *de novo* DNA methyltransferases Dnmt3a and Dnmt3b, but it lacks the catalytic activity (26). Numerous results indicated that this protein could function as an accessory factor to Dnmt3a and Dnmt3b. Indeed, it interacts with the catalytically competent methyltransferases, increases their activity at least *in vitro* (27), and stabilizes them as in the case of the Dnmt3a2 isoform (21). Dnmt3l interaction with Dnmt3a was characterized by analyzing the crystal structure of the complex confirming the formation of a tetramer including two Dnmt3l subunits, each interacting with Dnmt3a through their C-terminal domains (28). This structure is compatible with the binding of the N-terminal ATRX-Dnmt3-Dnmt3L domain of Dnmt3l with unmethylated lysine 4 of histone H3, which could favor the targeting of catalytically competent methyltransferases to specific chromatin domains (29). Furthermore, Dnmt3l also interacts with Ezr2, a subunit of the polycomb complex polycomb repressive complex 2, and this results in the protection of bivalent gene promoters from *de novo* methylation by Dnmt3a and Dnmt3b, thus keeping them competent for the following activation as part of specific differentiation programs (22). Although Dnmt3l KO mice have no defects at birth, they are sterile showing a clear germ line phenotype, thus indicating that Dnmt3l could be necessary for Dnmt3a-dependent methylation in gametogenesis (21). However, Dnmt3a deficiency is accompanied by undermethylation of DNA in the early steps of embryo development (21, 22, 23, 24, 25, 26, 27, 28, 29, 30, 31). Our results clearly indicate that Dnmt3l downregulation induced a phenotype similar to that observed in YAP KD cells. However, its OE in YAP KD cells failed to rescue the normal phenotype, thus indicating that there are other genes, whose expression is affected by YAP transient downregulation that contribute to the genesis of the observed phenotype.

Another gene whose behavior was altered in YAP KD cells is that transcribing for Eprn, a mouse-specific lncRNA. It was observed that Eprn is transiently upregulated upon the exit of ESCs from naive pluripotency and its suppression, by either gene KO or RNAi, was accompanied by a delayed downregulation of pluripotency-associated genes (19). One of the phenotypes found in Eprn KO cells is reduced methylation at the Nanog gene promoter, which mimics the decreased methylation observed in *Dnmt3a/b* gene KO cells (19). Thus, on the basis of our results, it is possible that in YAP KD ESCs, the decreased basal levels of Eprn and the absence of any Epnr induction upon ESC differentiation (Fig. 5*B*) have a negative effect upon the *de novo* genome methylation. Mammalian genomes produce thousands of lncRNAs, but their functions are in most cases still not definitively addressed. Numerous lncRNAs have regulatory roles in ESC (32), and many of them have a role in the regulation of the Hippo pathway (33). Less is known about the possible regulation of lncRNA gene expression by the Hippo pathway.

Although we demonstrated that the transcription of *Eprn* gene is under the control of YAP, which binds to a *cis* element is the second intron of the gene and that this binding is dependent on TEAD1/2, the mechanisms through which YAP downregulation causes a decrease of Dnmt3l could be indirect. The ChIP-Seq of YAP in undifferentiated ESCs showed a relatively small number of significant peaks, likely because of the relatively small fraction of undifferentiated ESCs where YAP is robustly expressed in the nucleus. As expected, we found numerous deregulated genes of well-known YAP direct targets, such as Cyr61, GADD45a, Wwc2 (for a complete list, see Table S2). Our ChIP-Seq assays did not show any evidence of binding of YAP in the proximity of *Dnmt3l* gene, so it is conceivable that its downregulation in YAP KD depends on an indirect mechanism, acting either at mRNA level and/or at protein level. TargetScan (34) did not find any conserved miRNA binding sites in the 3'UTR of Dnmt3l, but of course this does not exclude the possibility that Dnmt3l suppression was dependent on a miRNA-dependent mechanism.

## Experimental procedures

### Cell culture and transfection

E14Tg2a (BayGenomics) mouse ESCs were maintained on feeder-free and gelatin-coated plates in the following ESC medium: Glasgow minimum essential medium (Sigma) supplemented with 2 mM glutamine, 1 mM sodium pyruvate, 1× nonessential amino acids (all from Thermo Fisher Scientific), 0.1 mM β-mercaptoethanol (Sigma), 10% fetal bovine serum (HyClone Laboratories), and 10^3^ U/ml LIF (Merck).

Generation and culture of the α1tub–GFP and Mlc2v–GFP cell lines have been described previously (13, 14).

The pCAG–mYAP vector was generated by cloning the coding sequence of wt mouse YAP (from bac clone no.: MR226049-Origene) into the pCAG vector (13).

The pCAG-3xflag-mDnmt3L was generated by cloning the coding sequence of wt mouse DNMT3L (from pMX-Dnmt3l vector—Addgene) into the pCAG-3xflag vector (13). Transfections of siRNAs, shRNAs, pCAG-mYAP, and pCAG-3xflag-Dnmt3L plasmids were performed using Lipofectamine 2000 (Thermo Fisher Scientific) following the manufacturer's instructions. shRNAs form mouse pSM2 retroviral shRNAmir library (Open Biosystems) were as follows: 5'-TGC TGT TGA CAG TGA GCG AGC AGA CAG ATT CCT TTG TTA ATA GTG AAG CCA CAG ATG TAT TAA CAA AGG AAT CTG TCT GCG TGC CTA CTG CCT CGG A-3' for YAP KD; 5'-TGC TGT TGA CAG TGA GCG CTC GCT TGG GCG AGA GTA ATA GTG AAG CCA CAG ATG TAT TAC TCT CGC CCA AG CGA GTT GCG TGC CTA CTG CCT CGG A-3' for CONTROL (nonsilencing) KD.

Stealth siRNAs (Thermo Fisher Scientific; catalog no./ID: 1320001) were as follows: MSS238823 and MSS238824 for YAP; MSS211217 and MSS211218 for TEAD1; MSS278118 and MSS278119 for TEAD2; as well as MSS244161 and MSS285157 for DNMT3L. FlexiTube siRNAs (Qiagen; catalog no./ID: 1027417) for EPRN KD were as follows: SI05681977 and SI05681984. Stealth siRNA Negative Control Med GC Duplex (Thermo Fisher Scientific; catalog no./ID: 12935112 and 12935113) was used for negative controls.

### AP staining as well as neuroectodermal and mesodermal differentiation

For AP staining, ESCs were cultured at clonal density (30 cells/cm^2^). Cells were fixed in 10% cold neutral formalin buffer (10% formalin, 110 mM Na_2_HPO_4_, and 30 mM NaH_2_PO_4_ H_2_O) for 15 min and then rinsed in distilled water for 15 min. The staining was obtained by incubation for 45 min at room temperature with the following staining solution: 0.1 M Tris·HCl, 0.01% naphthol AS MX-PO4 (Sigma), 0.4% *N*,*N*-dimethylformamide (Sigma), 0.06% red violet LB salt (Sigma).

For neuroectodermal differentiation, ESCs were induced to differentiate either in monolayer (13), by placing 3 × 10^3^ ESCs per square centimeter in cell gelatin-coated cell culture plates, or by formation of SFEBs (15), by placing 1 × 10^6^ ESCs in 100 mm petri dishes, in the following differentiation medium: Glasgow minimum essential medium supplemented with 2 mM glutamine, 1 mM sodium pyruvate, 1× nonessential amino acids, 0.1 mM β-mercaptoethanol, and 10% KO serum replacement (Thermo Fisher Scientific). SFEBs indicate aggregates at 4 days (T4) of ESC differentiation unless noted otherwise.

ESC differentiation into mesoderm was induced by formation of embryoid bodies and has been described previously (15).

### RNA isolation, reverse transcription, and qPCR

qPCR has been previously described (35). In brief, total RNA was extracted by TriSure (Bioline), and first-strand complementary DNA was synthesized using Mu-MLV RT (New England BioLabs) according to the manufacturer's instructions. qPCR was carried out with the QuantStudio 7 Flex (Thermo Fisher Scientific) using Fast SYBR Green PCR Master Mix (Thermo Fisher Scientific). The housekeeping actin mRNA was used as an internal standard for normalization. Gene-specific primers used for amplification are listed in Table S5. qPCR data are presented as FCs relative to the indicated reference sample. mRNA expression levels were analyzed performing a comparative analysis using 2^−ΔΔCt^.

### Antibodies and Western blot analysis

Undifferentiated and differentiated ESCs were lysed in radioimmunoprecipitation assay buffer containing 150 mM sodium chloride, 1% NP-40, 0.5% sodium deoxycholate, 0.1% SDS, 50 mM Tris, pH 8.0, and protease inhibitor cocktail (Sigma–Aldrich), and analyzed by Western blot. The following primary antibodies were used: rabbit anti-Yap (1:1000; D8H1X; Cell Signaling Technology), rabbit anti-Dnmt3l (1:1000; E1Y7Q; Cell Signaling Technology), mouse anti-Vinculin (1:1000; G11; Santa Cruz Biotechnology), and mouse anti-Flag (1:2000; Sigma). Western blots were developed with an ECL system (BioRad) using the following horseradish peroxidase–conjugated antibodies: anti-rabbit IgG (1:10,000), antimouse IgG (1:5000; both from Amersham Pharmacia Biotech).

### FACS analysis

Analysis of DNA content by propidium iodide incorporation to evaluate cell death was performed in permeabilized cells by flow cytometry. ESCs were dissociated and collected, washed in PBS, and resuspended in a solution containing 0.1% sodium citrate w/v, 0.1% TritonX-100 v/v, and 50 mg/ml propidium iodide (Sigma). After incubation at 4 °C for 30 min in the dark, cell nuclei were analyzed with a FACS Accuri C6 (Becton Dickinson). Cellular debris was excluded from the analysis by raising the forward scatter threshold, the DNA content of the nuclei was registered on a logarithmic scale, and the percentage of the elements in the hypodiploid region was calculated.

### Immunostaining and microscopy

For immunofluorescence analysis, ESCs were fixed, permeabilized, and incubated with primary antibodies and an appropriate secondary antibody (13). Nuclei were counterstained with 4',6-diamidino-2-phenylindole (1:5000; Calbiochem). Sectioned SFEBs were obtained and stained as previously described (15). The following primary antibodies were used: anti-Yap (1:300; NB110-58358; Novus Biological) and anti-βIII tubulin (1:400; Sigma). Alexa Fluor 594 or 488 secondary antibodies were used (1:400; Thermo Fisher Scientific). Cells were visualized using an inverted microscope (Leica Microsystems), and the images were captured with a digital camera (DFC365 FX; Leica Microsystems) using LAS-AF software (Leica Microsystems).

Confocal images were acquired with LSM510META microscope (Carl Zeiss GmbH) using LSM510 software (Zeiss). After acquisition, the images were color corrected using the brightness, contrast, and color-balance commands applied to every pixel in each image.

### RNA-Seq and analysis

Total RNAs from three independent samples each for YAP KD, CTR KD, YAP OE and CTR OE were extracted by TriSure (Bioline) and subjected to high-throughput sequencing with Illumina Genome Analyzer platform (Illumina). Reads have been mapped using STAR (36) on the mm10 reference genome using standard parameters. The RefSeq curated transcripts annotation downloaded from the University of California Santa Cruz (UCSC) Genome Browser database (37) was used as a reference to quantify expression through RSEM (38). Differential expression analysis was carried out using the edgeR package (39).

### Chromatin immunoprecipitation

For ChIP-Seq analysis, ESCs were crosslinked with 1% formaldehyde for 10 min at room temperature, and formaldehyde was then inactivated by the addition of 125 mM glycine. Cells were lysed, and the chromatin was sonicated to an average DNA fragment length of 200 to 500 bp. Soluble chromatin extracts were immunoprecipitated using the rabbit polyclonal anti-Yap (NB110-58358; Novus Biological) or rabbit IgG (Abcam) as control. ChIP-Seq library preparation was obtained by using the TruSeq ChIP Sample Prep Kit (Illumina) Then samples from two independent experiments were subjected to high-throughput sequencing with Illumina Genome Analyzer platform (Illumina). ChIP-Seq data were processed by Galaxy tools (40). Briefly, reads were mapped against the *Mus musculus* genome (UCSC, mm9) using bowtie software (version 0.9.9.1) with parameters -v 2 -a -m 100, tracking up to 100 best alignment positions per read and allowing at most two mismatches. Each alignment was weighted by the inverse of the number of hits. All quantifications were based on weighted alignments. Clusters of ChIP-Seq read alignments were identified employing MACS software (version 1.3.7.1). For ChIP-qPCR, samples were prepared as previously described (41). Supernatant obtained without antibody was used as an input control. qPCR analyses were performed by using the QuantStudio 7 Flex (Thermo Fisher Scientific) and Fast SYBR Green PCR Master Mix (Thermo Fisher Scientific). Primers used for ChIP–qPCR are listed in Table S5. The amount of precipitated DNA was calculated relative to the total input chromatin and expressed as the fold enrichment relative to total input according to the following formula: fold enrichment = 2{Delta}Ct × 10, where {Delta}Ct = Ct(input) − Ct(immunoprecipitation), where Ct refers to cycle threshold.

### BS-Seq and analysis

Genomic DNA from two independent experiments each for YAP KD T0, YAP KD T4, CTR KD T0, and CTR KD T4 was extracted and purified by QIAamp DNA Kit (QIAGEN) according to the manufacturer's instructions. Reads have been quality trimmed using Trim Galore 0.6.5 (http://www.bioinformatics.babraham.ac.uk/projects/trim_galore/). Trimmed reads have been mapped on the mm9 reference genome (after *in silico* bisulphite conversion) using the BS-Seq alignment function of Bismark (42) with default options. Calling of methylated cytosines has been made using the methylation extractor utility of Bismark. DMRs have been identified by means of the methylKit R package (43) using a tiling window of length 250 bp and step 125 bp. The window size was set to 250 bp because this value is close to the median length (240 bp) of the SureSelectXT Methyl-Seq Target Enrichment regions. Positions covered by less than six reads were excluded from the analysis. DMRs have been annotated using the genomation R package (44) on the mm9 RefSeq gene annotation downloaded from the UCSC Genome Browser database (38).

### Statistical analysis

The number of biological replicates of each experiment is indicated in the legends to the figures. The means of at least two independent experiments were used to calculate SEM or SD and to perform statistical analysis (when appropriate). All *p* values were calculated by Student's *t* test, using a two-tailed test and paired samples.

## Data availability

All sequencing data have been deposited in the public repository Gene Expression Omnibus database (GSE157707).

## Conflict of interest

The authors declare that they have no conflicts of interest with the contents of this article.
