# Supplementary material for: YAP contributes to DNA methylation remodeling upon mouse embryonic stem cell differentiation
Source: J Biol Chem. 2020 Dec 6;296:100138. doi: 10.1074/jbc.RA120.015896 (PMC7948423; doi:10.1074/jbc.RA120.015896)
Supplement: Supplementary file 1 [file mmc1.pdf]

Supporting Figure S1

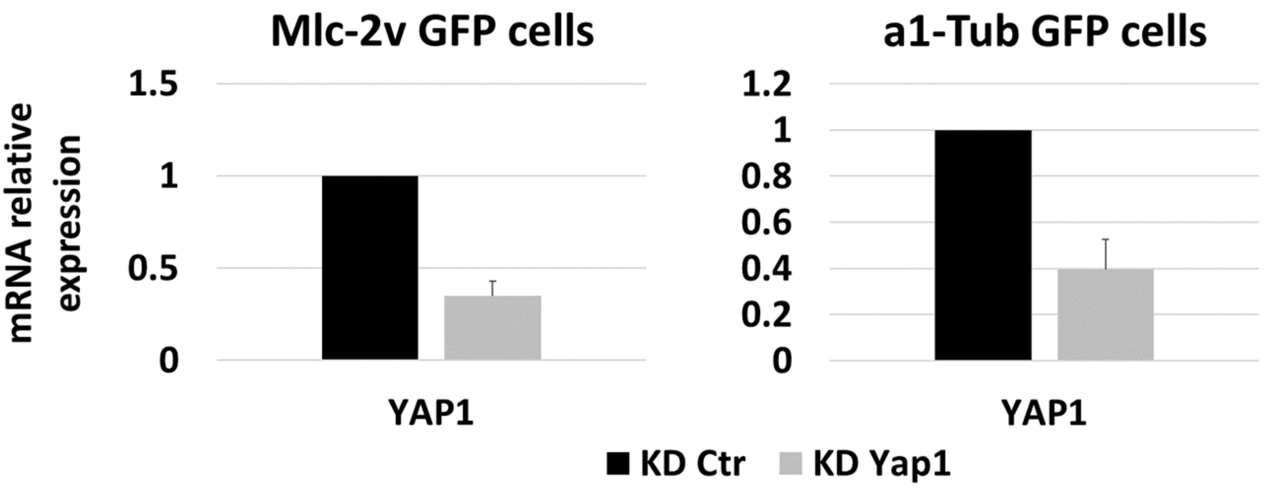

Figure S1: qPCR for validation of YAP KD in Mlc2v- and a1T- GFP cells.

Supporting Figure S2

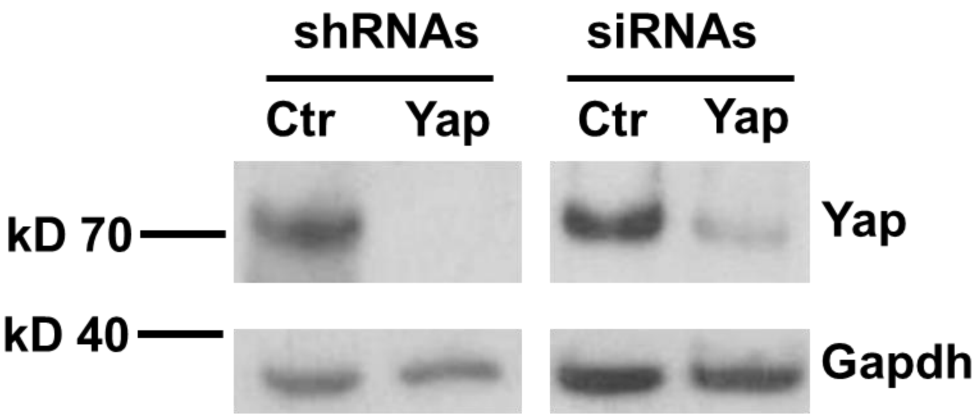

Figure S2-A: Western Blot for validation of YAP KD in ESCs by siRNAs or shRNAs transfection.

Supporting Figure S3

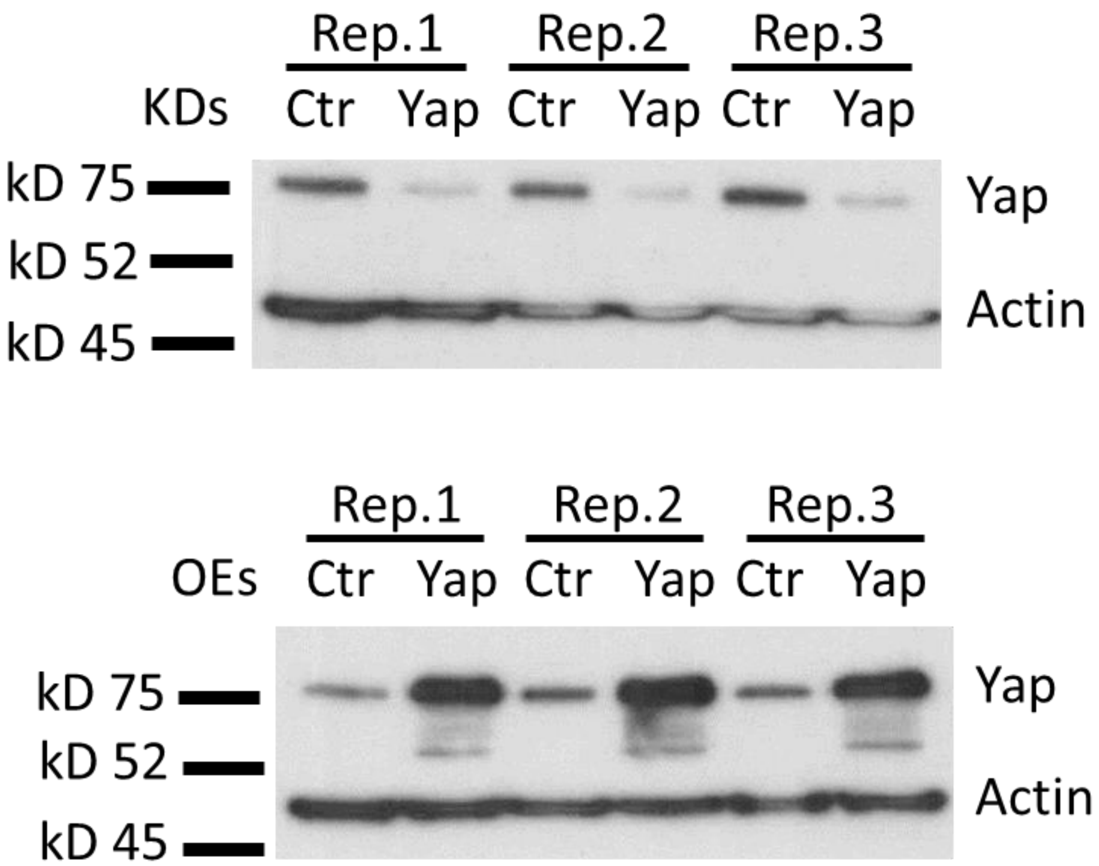

Figure S3: Western Blot for validation of YAP KD and OE in biological triplicates analyzed by RNA-seq

Supporting Figure S4

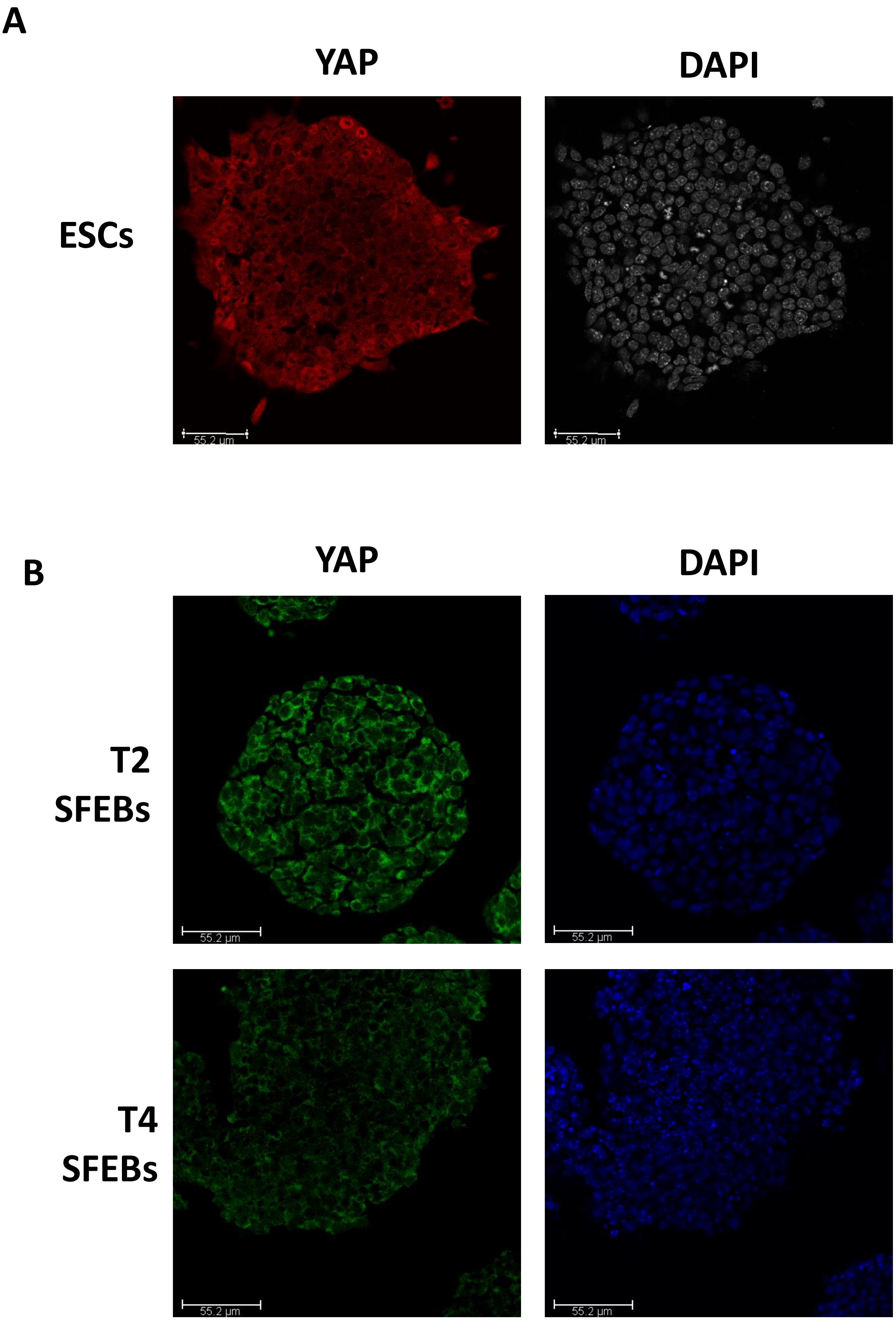

Figure S4: Representative immunostaining of YAP in undifferentiated ESCs (A) or differentiated SFEBs at T2 and T4 (B) Scale bar: 55 µm.

Supporting Figure S5

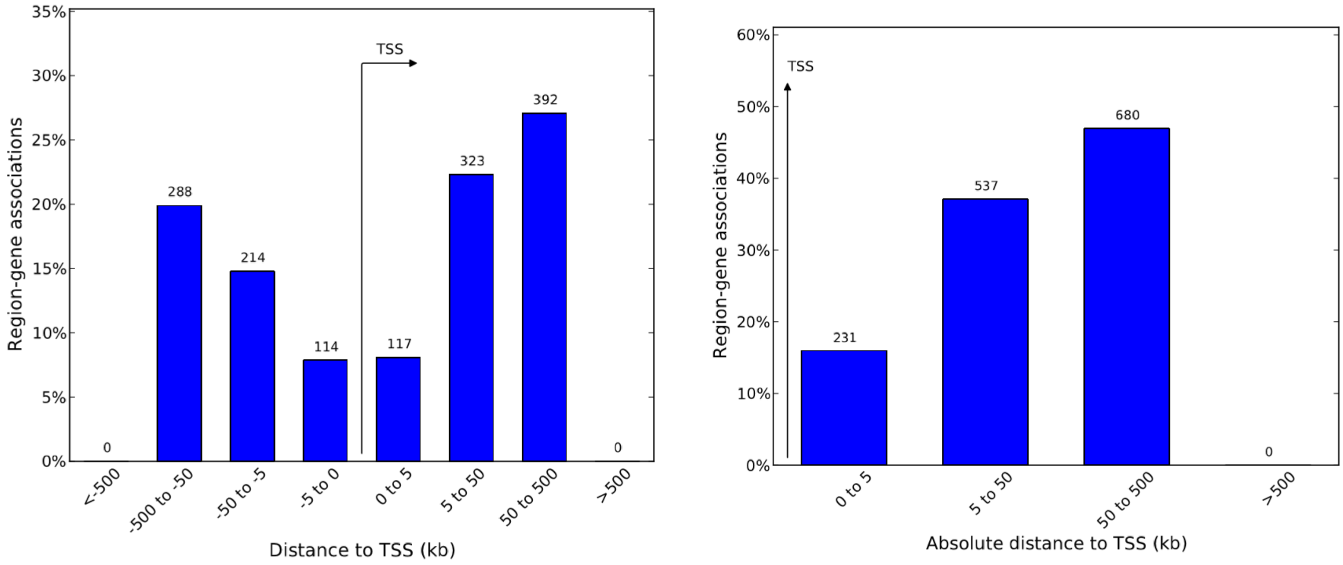

Figure S5-A : Distribution of YAP peaks with respect to TSS

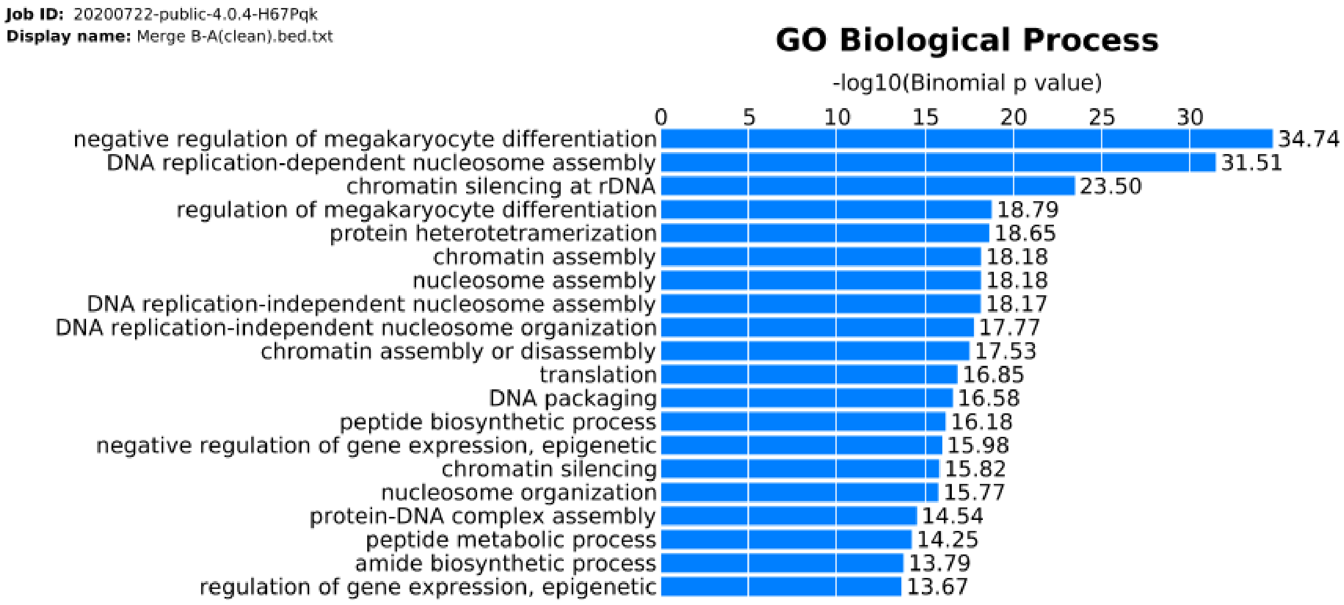

Figure S5-B : Functional enrichment analysis showing the significant (FDR<0.05) clusterization of putative target genes in specific GO BPs.

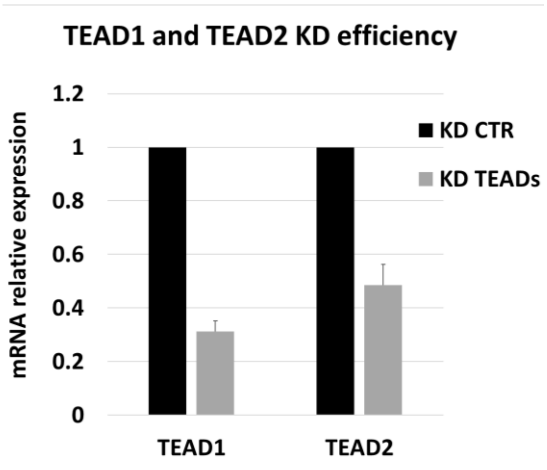

Figure S5-C : qPCR for validation of TEAD1 and TEDA2 KD in E14Tg2a cells.

Supporting Figure S6

A

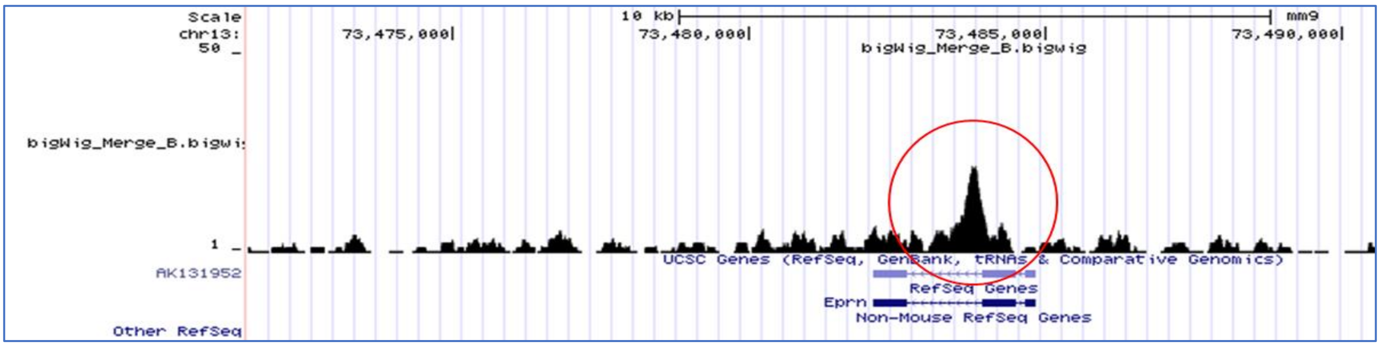

Figure S6-A: Location of the ChIP-seq peak mapping on Epnr gene by Genome Browser.

Supporting Figure S6 (continuing)

B

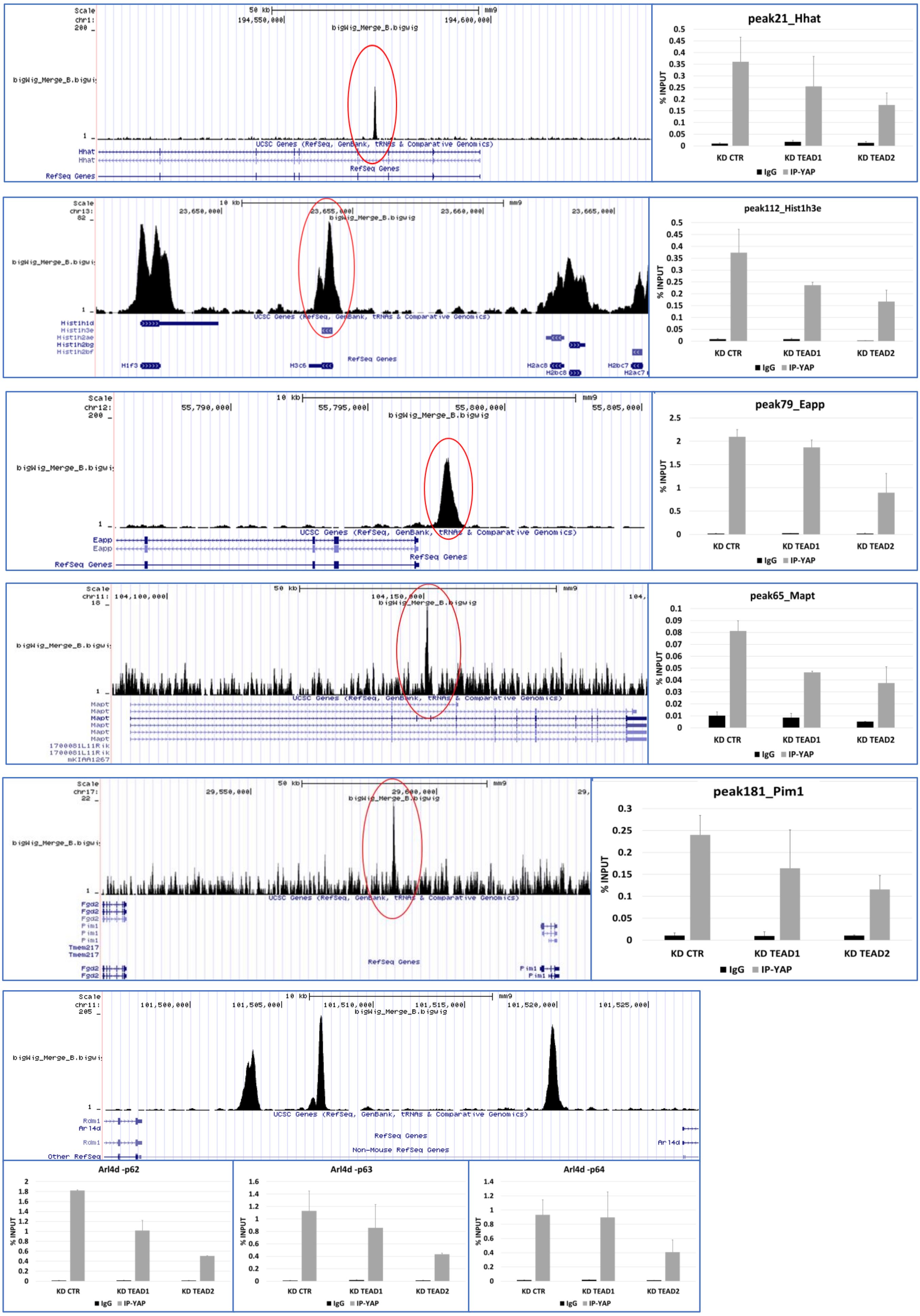

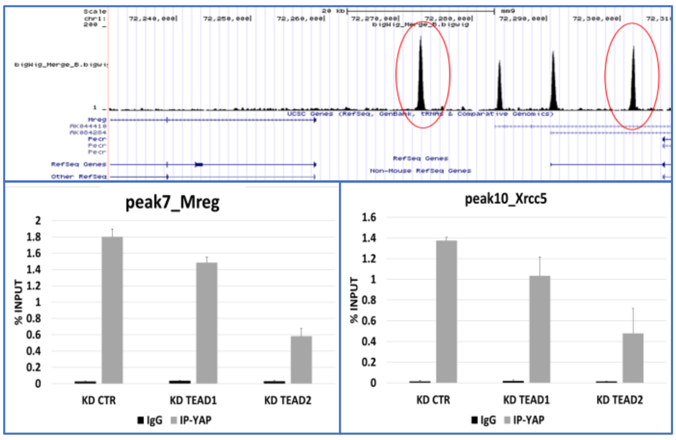

Figure S6-B: Location of the ChIP-seq peak mapping on validated gene by Genome Browser.

Supporting Figure S7

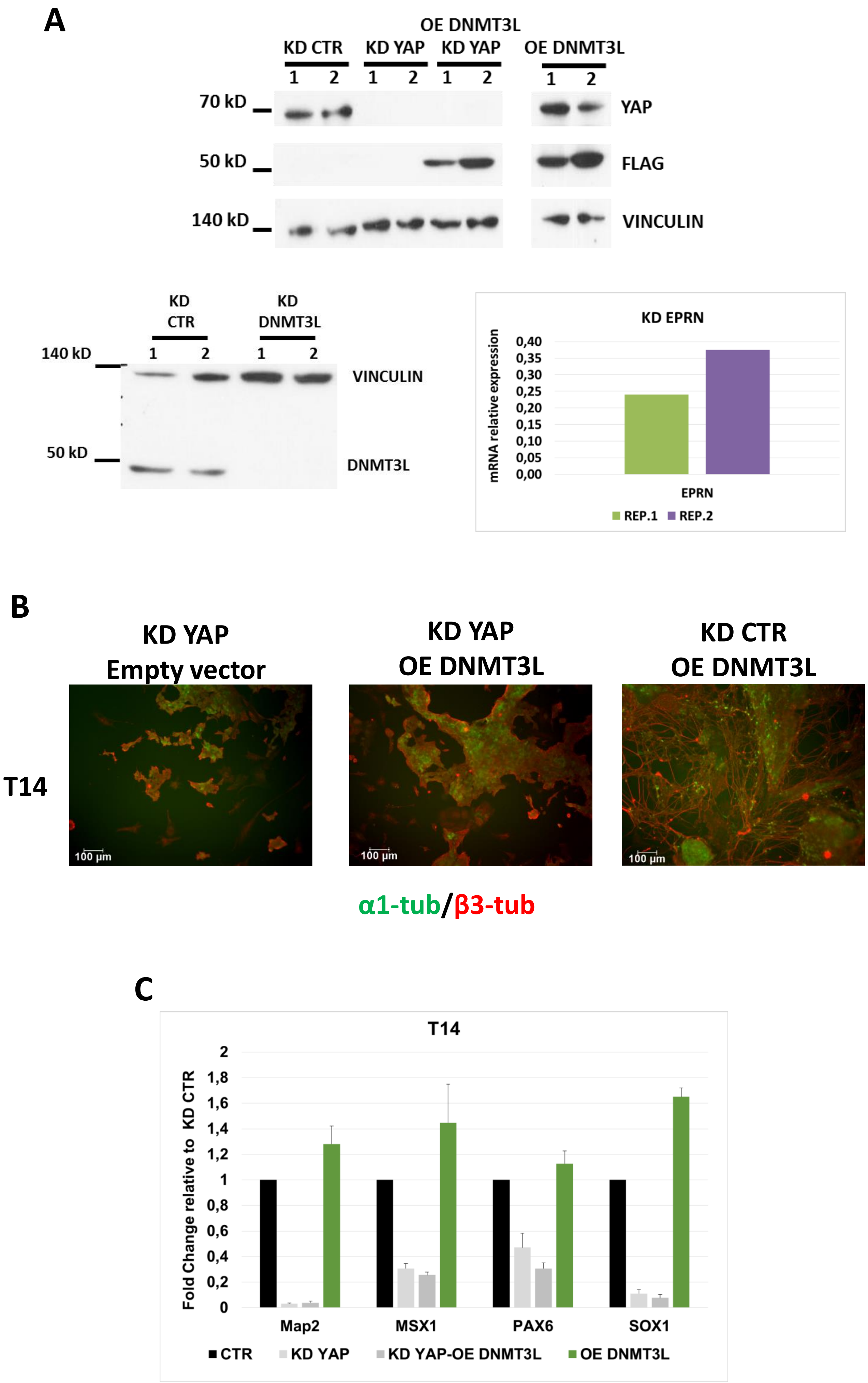

Figure S7: A) Western Blot and qPCR for validation of EPRN KD, YAP KD, DNMT3L KD and DNMT3L OE in ESCs. Biological duplicates (1 and 2) are shown. B) Representative immunostaining of neural marker β3-tubulin (red) and α1-tubulin (green) at final stage (T14) of differentiation. Scale bar: 100µM. C) qPCR analysis of neuronal marker gene expression upon differentiation of YAP KD, with or without DNMT3L OE, and DNMT3L OE cells. Data are shown as Fold Changes with respect to KD CTR/empty vector cells.
